# Null Effect of Transcranial Static Magnetic Field Stimulation over the Dorsolateral Prefrontal Cortex on Behavioral Performance in a Go/NoGo Task

**DOI:** 10.3390/brainsci11040483

**Published:** 2021-04-11

**Authors:** Tatsunori Watanabe, Nami Kubo, Xiaoxiao Chen, Keisuke Yunoki, Takuya Matsumoto, Takayuki Kuwabara, Toru Sunagawa, Shota Date, Tatsuya Mima, Hikari Kirimoto

**Affiliations:** 1 Department of Sensorimotor Neuroscience, Graduate School of Biomedical and Health Sciences, Hiroshima University, Hiroshima 734-8553, Japan; twatan@hiroshima-u.ac.jp (T.W.); e.orinoco26@gmail.com (N.K.); d185984@hiroshima-u.ac.jp (X.C.); d205546@hiroshima-u.ac.jp (K.Y.); d203652@hiroshima-u.ac.jp (T.M.); m203434@hiroshima-u.ac.jp (T.K.); 2 Japan Society for the Promotion of Science, Tokyo 102-0083, Japan; 3 Department of Analysis and Control of Upper Extremity Function, Graduate School of Biomedical and Health Sciences, Hiroshima University, Hiroshima 734-8553, Japan; torusuna@hiroshima-u.ac.jp (T.S.); sdate@hiroshima-u.ac.jp (S.D.); 4 Graduate School of Core Ethics and Frontier Sciences, Ritsumeikan University, Kyoto 603-8577, Japan; t-mima@fc.ritsumei.ac.jp

**Keywords:** transcranial static magnetic field stimulation, non-invasive brain stimulation, cognitive function, dorsolateral prefrontal cortex, response inhibition, Go/NoGo task

## Abstract

The purpose of this pilot study was to investigate whether transcranial static magnetic field stimulation (tSMS), which can modulate cortical excitability, would influence inhibitory control function when applied over the dorsolateral prefrontal cortex (DLPFC). Young healthy adults (*n* = 8, mean age ± SD = 24.4 ± 4.1, six females) received the following stimulations for 30 min on different days: (1) tSMS over the left DLPFC, (2) tSMS over the right DLPFC, and (3) sham stimulation over either the left or right DLPFC. The participants performed a Go/NoGo task before, immediately after, and 10 min after the stimulation. They were instructed to extend the right wrist in response to target stimuli. We recorded the electromyogram from the right wrist extensor muscles and analyzed erroneous responses (false alarm and missed target detection) and reaction times. As a result, 50% of the participants made erroneous responses, and there were five erroneous responses in total (0.003%). A series of statistical analyses revealed that tSMS did not affect the reaction time. These preliminary findings suggest the possibility that tSMS over the DLPFC is incapable of modulating inhibitory control and/or that the cognitive load imposed in this study was insufficient to detect the effect.

## 1. Introduction

Transcranial static magnetic field stimulation (tSMS) is one of non-invasive brain stimulation (NIBS) tools that can modulate brain activity as well as motor and cognitive functions. Since Oliviero and colleagues reported a reduction of motor cortical excitability after application of tSMS over the motor cortex in 2011 [1], a relatively larger number of studies have been conducted to investigate the effectiveness of this new tool. For example, tSMS has been shown to reduce somatosensory-evoked potentials when applied over the sensorimotor cortex [2,3,4], and also to increase alpha-band power when applied over the parietal or occipital cortex [5,6]. Furthermore, recent studies revealed changes in cortical activity in brain regions apart from the site of stimulation [7,8,9,10]. With regard to behavioral parameters, tSMS has been demonstrated to impair performance of difficult reaction time task [5], postural adjustment task [11], and force-matching task [12] when it was applied over the occipital cortex, supplementary motor area, and motor cortex, respectively. Moreover, tSMS has an ability to modulate symmetry judgements with the temporal cortex stimulation [13] and to enhance offline learning with the motor cortex stimulation [14], although the enhancement of offline learning by tSMS was not evident in another study [15]. Collectively, even though non-significant changes in sensorimotor cortical excitability by tSMS found in some previous studies should be acknowledged [16,17], tSMS has the potential to induce modulations of various human functions.

Inhibitory control is an important component of executive functions, enabling one to reduce impulsive behavior and to suppress inappropriate responses and actions. When this function is impaired, various kinds of behavioral problems, such as food and smoking urges [18,19], drug and alcohol abuse [20], and hasty decision making [21,22,23], can occur. From this perspective, a number of studies have examined whether impaired inhibitory control can be rehabilitated using cognitive training or NIBS tools. Particularly, repetitive transcranial magnetic stimulation (rTMS) and transcranial direct current stimulation (tDCS), which either increase or decrease the cortical excitability depending on the stimulation setting, have received a great attention during a past decade as cognitive training has been shown to have a small effect on impulsive behavior in a recent meta-analysis [24]. For instance, excitatory high-frequency rTMS over the left dorsolateral prefrontal cortex (DLPFC), one of brain regions in an inhibitory control network [25], increased accuracy in a Go/NoGo task that assesses proactive inhibitory control in alcohol dependent individuals [26]. Similarly, excitatory anodal tDCS over the left DLFPC increased accuracy in a Go/NoGo task in individuals with major depressive disorder [27]. Interestingly, inhibitory cathodal tDCS over the left DLPFC also increased accuracy in the same task in students with attention deficit hyperactivity disorder (ADHD) [28]. On the other hand, there are also studies reporting non-significant effects of these NIBS techniques on inhibitory control function. For example, Go/NoGo task performance was found not to be affected by anodal tDCS over the left DLPFC in individuals with schizophrenia [29] and with ADHD [30,31]. Furthermore, high-frequency rTMS over the right DLPFC has been demonstrated not to influence Go/NoGo task performance in individuals with alcohol use disorders [32,33]. At present, no firm conclusion has been made as to whether these techniques are effective in treating inhibitory control function of individuals with clinical conditions, and continuous investigation on this topic is warranted to evaluate the clinical use of NIBS techniques, including tSMS.

Accordingly, the purpose of this study was to investigate the effect of tSMS over the DLPFC on behavioral performance in a Go/NoGo task. Static magnetic fields (SMFs) by tSMS have been demonstrated to reduce the cortical excitability [1]. Although the exact mechanism underlying this reduction has not been fully understood, it is proposed that SMFs induce reorientation of membrane phospholipids, which results in deformation of ion channels and their function [34,35,36]. Since performance of Go/NoGo task was found to be impaired by cathodal tDCS, which reduces the cortical excitability, irrespective of brain side in healthy adults [37,38], we hypothesized that tSMS over the left/right DLPFC would similarly impair performance. If tSMS is capable of modulating the inhibitory control function and thus the DLPFC, it may be used to correct asymmetric activity of the frontal lobes (i.e., hypoactivation in one side and hyperactivation in the other side) observed in individuals with such clinical conditions as major depressive disorder and impulsive behavior. 

## 2. Materials and Methods

### 2.1. Participants

Eight healthy adults (mean age ± SD = 24.4 ± 4.1, six females) participated in this study. They were university students or graduates. Exclusion criteria included a history of neurological and psychiatric disorders. None of them had metal implants or were under medical treatment for any condition. They were all right-handed as confirmed by Edinburgh Handedness Inventory and had normal or corrected-to-normal vision.

### 2.2. Procedure

**Figure 1:**
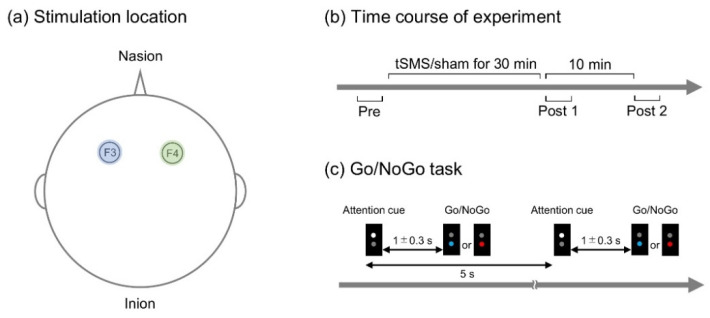
Experimental task and procedure. TSMS or sham stimulation was applied over F3 or F4 of international 10–20 system (**a**). The performance of Go/NoGo task was evaluated before (pre), immediately after (post 1), and 10 min after (post 2) tSMS/sham stimulation (**b**). The participants made a response to a blue light and withheld the response to a red light (Go/NoGo task) (**c**).

A cylindrical NdFeB neodymium magnet (diameter, 50 mm; height, 30 mm) with a surface magnetic flux density of 534 mT, maximum energy density of 49 MGOe, and a strength of 862 N (88 kgf) nominal value (NeoMag, Ichikawa, Japan) was used for tSMS. A non-magnetic stainless-steel cylinder of the same size and weight was used for sham stimulation. With the aid of an arm-type lightning stand (C-stand, Avenger, Cassola, Italy), the magnet or sham device was placed over F3 or F4 of international 10–20 system of electrode placement (Figure 1a). The stimulation site for sham stimulation (F3 or F4) was randomized among the participants [13].

Each of these stimulations was given to all the participants in a random order on different days (at least one day apart), and the stimulation was blinded to the participants (single-blind study). The participants performed a Go/NoGo task before (pre), immediately after (post 1), and 10 min after (post 2) the stimulation (Figure 1b). The protocol was the same for tSMS and sham. 

### 2.3. Go/NoGo Task

A custom-made illumination device with light emitting diodes (LED) (4 Assist, Tokyo, Japan) was used to present visual stimuli (Figure 1c). Trials started with a white LED stimulus (attention cue of 150 ms), which was followed by either a blue or red LED stimulus (150 ms) with an interstimulus interval of random duration between 1000 and 1300 ms. The blue LED stimulus served as a target signal (Go), and the red LED stimulus served as a non-target signal (NoGo). The intertrial interval was 5 s. Similar to previous studies [39], 10 target and 10 non-target signals were presented randomly in one block of trials.

The participants sat on a chair comfortably 100 cm in front of the custom-made illumination device with the pronated right forearm resting on a flat armrest. They were instructed to extend the right wrist as fast as possible when the target stimulus was presented and to withhold the response when the non-target stimulus was presented. Responses to the stimuli were evaluated using electromyogram (EMG) activity recorded from the right wrist extensor muscles.

### 2.4. EMG

EMG was recorded from the right wrist extensor muscles. The disposable Ag/AgCl electrodes were placed over the right wrist extensor muscles (one-third the distance from the olecranon to the distal head of the ulna) with an interelectrode distance of approximately 2 cm. The signal was amplified (FSE-DEMG1, 4 Assist, Japan), filtered between 5 and 1000 Hz, and digitized at 10k Hz using an A/D converter (PowerLab, AD Instruments, Sydney, Australia).

### 2.5. Data Analysis

The raw EMG signal was full-wave rectified and averaged over 500 ms pre-stimulus interval. The activation onset of the right wrist extensor muscles was defined as a time point at which the rectified EMG amplitude exceeded 3SD from the pre-stimulus average. Responses to non-target signals and non-responses to target signals were counted as errors (false alarm and missed target detection).

### 2.6. Statistical Analysis

R (R development team) was used for statistical analysis. We performed a two-way repeated measures analysis of variance (ANOVA) to examine the effect of stimulation (F3, F4, and sham) and time (pre, post 1, and post 2) on reaction times after confirming that they were normally distributed (Shapiro–Wilk test). Greenhouse–Geisser correction was applied when the sphericity assumption was violated. No statistical test was performed for erroneous responses as there were only five erroneous responses in total (0.003%). Significant level was set at 0.05.

## 3. Results

**Figure 2:**
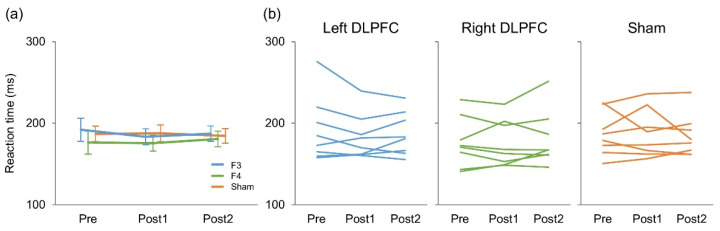
Mean (**a**) and individual (**b**) reaction times during Go/NoGo task.

There were five erroneous responses in total (0.003%), and 50% of the participants made no errors at all (Table 1). Figure 2 presents reaction time results. A two-way repeated measures ANOVA revealed no significant main effect of stimulation (F_(2,14)_ = 2.47, *p* = 0.121, η^2^ = 0.024) or time (F_(2,14)_ = 0.33, *p* = 0.724, η^2^ = 0.0018) on reaction times. There was no significant interaction between them (F_(4,28)_ = 0.94, *p* = 0.46, η^2^ = 0.0066). 

To explore these reaction-time findings in depth, we additionally performed a Bayesian two-way repeated measures ANOVA on the same data using JASP (JASP Team (2020). JASP (Version 0.14.1) (Windows 10)) with the JASP’s default prior. This analysis tests whether the experimental data provide stronger evidence for the null or alternative hypothesis, and a Bayes Factor (BF_10_) < 1/3 indicates greater support for the null while a BF_10_ > 1 indicates greater support for the alternative [40]. We found that the BF_10_ for the main effects of stimulation and time were 3.59 and 0.15, respectively, and that the BF_10_ for the interaction between these factors was 0.11. The post-hoc comparisons for the stimulation effect based on the default *t*-test with a Cauchy (0, r = 1/sqrt(2)) prior provided evidence indicating that the reaction time was faster in F4 than F3 (BF_10_ = 6.56, posterior odds = 3.86) and sham stimulation condition (BF_10_ = 5.61, posterior odds = 3.30) regardless of the time point of measurement (i.e., pre, post 1, and post 2).

Moreover, to ensure the non-significant interaction, we performed equivalence testing, which can differentiate true negative results from underpowered studies [41,42,43] using R’s equivalence package (https://cran.r-project.org/web/packages/equivalence/index.html, accessed on 10 April 2021). Equivalence testing was conducted with a two one-sided test procedure in a pair-wise fashion between different time points of measurement (i.e., pre, post 1, and post 2) for each stimulation condition. As the effect of NIBS on reaction time in proactive inhibition tasks has been reported to be approximately 20–30 ms [44,45,46], an equivalent threshold of 20 ms was considered to be the minimum relevant difference. We found that the difference in reaction time between pre and post 1, between pre and post 2, and between post 1 and post 2 was significantly equivalent to zero for all the stimulation conditions (*p* < 0.05). 95% confidence intervals are provided in Table 2.

**Figure 3:**
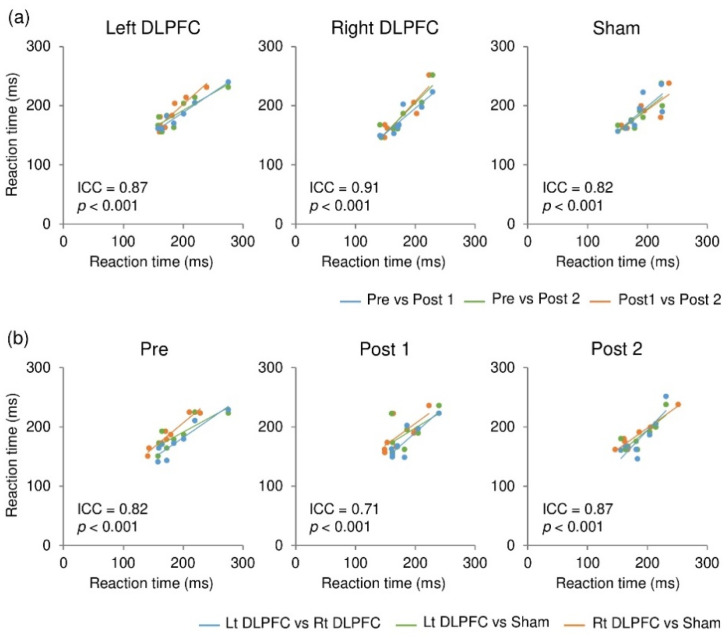
Consistency of reaction times within individuals over time (**a**) and across different stimulation conditions (**b**).

To assess stability (individual consistency) of the reaction time over time and across different stimulation conditions, intraclass correlation coefficients (ICC) with a two-way model and absolute agreement were calculated using R for each stimulation condition as well as for each time point of measurement (i.e., pre, post 1, and post 2). The analysis showed that the reaction times were consistent within participants over time, with ICC values of 0.87 (*p* < 0.001) for F3 (left DLPFC), 0.91 (*p* < 0.001) for F4 (right DLPFC), and 0.82 (*p* < 0.001) for sham (Figure 3a). Additionally, they were consistent within participants across different stimulation conditions, with ICC values of 0.82 (*p* < 0.001) for pre, 0.71 (*p* < 0.001) for post 1, and 0.87 (*p* < 0.001) for post 2 (Figure 3b). These ICC values indicate that agreement was substantial (0.60 < ICC ≤ 0.8) or almost perfect (ICC ≤ 0.81) [47].

## 4. Discussion

This study examined the effect of tSMS over the DLPFC on response inhibition using a Go/NoGo task. We found that the stimulation affected neither erroneous response nor reaction time. Furthermore, the reaction time was almost perfectly/substantially consistent within individuals over time and across different stimulation conditions. Our findings suggest the possibility that tSMS over the DLPFC is incapable of modulating inhibitory control function and/or that the cognitive load imposed during the task used in this study was insufficient to detect the tSMS effect. 

In a Go/NoGo task, a bias toward response execution can result in increased erroneous responses, while a bias toward response inhibition can result in lengthened reaction times [48]. Thus, when the number of erroneous responses is not affected by NIBS, reaction time to the target signal can be expected to change (speed-accuracy tradeoff). Indeed, previous studies have reported that NIBS over the DLPFC did not affect accuracy but reduced reaction time in cognitive tasks in healthy individuals [49,50,51]. However, in the present study traditional and Bayesian ANOVA both revealed no significant difference in either accuracy or reaction time between different time points of measurement, indicating that tSMS over the DLPFC has no effect on the performance of Go/NoGo task. The subsequent equivalent testing results further support this argument.

There are several explanations that account for our findings. First, tSMS could have not been strong enough to modulate the DLPFC. In previous studies, we and the other group confirmed that SMF of NdFeB magnet decreases in inverse proportion to the square of the distance but is strong enough to have biological effects on most surface cortical sites (110–190 mT at 2–3 cm from the magnet surface) [2,52]. Additionally, a reduction of cortical excitability by around 20–25% has been continuously reported in single-pulse TMS and somatosensory-evoked potential experiments [1,2]. However, as mentioned in a review about the effect of tDCS on cognitive function [50], the effect of NIBS (including tSMS) on the prefrontal cortex including the DLPFC may be different from that on the sensorimotor cortex. Furthermore, the effect of NIBS on cognitive function could be in a proportional relationship with its strength. Thus, it is conceivable that SMF of NdFeB magnet is strong enough to modulate the sensorimotor cortex but not the DLPFC, and it would be interesting to investigate whether the larger NdFeB magnet has the modulatory effect on the DLPFC in future studies. As an alternative hypothesis, there is a possibility that tSMS affected brain regions distant from the stimulation site since tSMS applied over the DLPFC has been reported to modulate cortical oscillations not in the DLPFC but in the occipital and frontoparietal cortices [9]. Further studies are warranted to better understand the relationship between tSMS and cognitive functions and their associated brain networks. 

Second, it is possible that the task used in the present study was too easy for our participants (i.e., healthy young adults) and did not impose enough cognitive load to recruit the DLPFC strongly. This assumption is supported by our finding of only five erroneous responses in total, and from this view it is reasonable to consider that the DLPFC that is only weakly activated in the first place is unlikely to be modulated by tSMS. According to four recent meta-analyses about the effect of tDCS over the DLPFC on cognitive function [49,50,53,54], accuracy and/or reaction time can be improved with anodal tDCS in neuropsychiatric patients; however, there was a disagreement on these effects in healthy individuals. This potentially indicates that baseline activity level of the DLPFC is one of factors influencing the effect of NIBS on cognitive function. That is, when it is high, it can be lowered using NIBS, and vice versa. With respect to tSMS, response time and erroneous response during a choice reaction time task have been reported to be unaffected by two hours of tSMS over the occipital cortex in healthy adults [55]. Further, a previous study using a visual search task, during which study participants searched for a target in an array containing distractors, demonstrated that an increase in reaction time by tSMS over the occipital cortex occurred selectively in the condition with high difficulty [5]. Therefore, the effect of tSMS over the DLPFC on cognitive function may be apparent with a task that imposes higher cognitive load than the Go/NoGo task used in the present study. 

Third, the site of stimulation may not have been appropriate to modulate the performance of Go/NoGo task. At the level of neural processes, inhibitory control function is mediated by a prefrontal-basal ganglia circuit [56,57,58,59] and has been reported to be carried out by two separate networks, one with the DLPFC and the other with the inferior frontal cortex (IFC) [60]. In previous studies, NIBS applied over the IFC was found to influence reactive inhibitory control, that is, to stop already initiated response [61,62] but not proactive inhibitory control [61]. Furthermore, NIBS over the DLPFC was revealed to impact the proactive inhibitory control in such task as Go/NoGo [37,63] and Stroop tasks [64]. From these evidences, the DLPFC and IFC are assumed to be involved in proactive and reactive inhibitory control, respectively. On the other hand, some previous studies provided evidence of proactive inhibitory control being influenced by tDCS over the IFC [46,65]. Therefore, although we think it unlikely, performance of Go/NoGo task may have been affected by tSMS over the IFC. 

The limitation of this study was the small sample size. Given the equivalence testing results and the almost perfect/substantial consistency of reaction time within participants over time and across different stimulation conditions, increasing the sample size seems unlikely to change our findings. On the other hand, the BF_10_ supporting the null hypothesis in our Bayesian analysis is categorized as moderate evidence according to Jeffreys’s scale [40]. Furthermore, there are a number of studies reporting interindividual variability in response to NIBS [66,67]. Therefore, further studies with more representative sample sizes are warranted to advance the field. 

## 5. Conclusions

In conclusion, this study suggests two possibilities: (1) tSMS over the DLPFC is incapable of modulating inhibitory control and (2) the cognitive load imposed by the current Go/NoGo task was insufficient to detect the tSMS effect. Further studies are necessary to investigate whether cognitive function is modulated by tSMS in other cognitive tasks or when applied to the other brain regions, to advance our understanding of the effectiveness of this NIBS as a clinical tool.

## Figures and Tables

**Table 1:** The number of erroneous responses in each task session (five in total).

Erroneous Responses								
Left DLPFC			Right DLPFC			Sham		
pre	post 1	post 2	pre	post 1	post 2	pre	post 1	post 2
1	0	0	1	1	1	1	0	0

**Table 2:** 95% confidence intervals from equivalence testing.

Left DLPFC		
Pre vs. Post 1	Pre vs. Post 2	Post 1 vs. Post 2
(−0.99, 18.44)	(−9.25, 18.80)	(−11.16, 3.26)
**Right DLPFC**		
Pre vs. Post 1	Pre vs. Post 2	Post 1 vs. Post 2
(−7.33, 8.90)	(−13.47, 4.55)	(−14.45, 3.96)
**Sham**		
Pre vs. Post 1	Pre vs. Post 2	Post 1 vs. Post 2
(−13.66, 11.88)	(−7.53, 12.36)	(−7.91, 14.52)

## Data Availability

Data available on request.
